# Effect of Ethnicity, Dietary Intake and Physical Activity on Plasma Adiponectin Concentrations Among Malaysian Patients with Type 2 Diabetes Mellitus

**DOI:** 10.5812/ijem.8298

**Published:** 2013-07-01

**Authors:** Koo Hui Chin, Daniel Robert Sathyasurya, Hazizi Abu Saad, Hamid Jan B Jan Mohamed

**Affiliations:** 1 Nutrition Program, School of Health Sciences, Universiti Sains Malaysia, Malaysia, Malaysia; 2 Department of Nutrition and Dietetics, Faculty of Medicine and Health Sciences, Universiti Putra Malaysia, Malaysia

**Keywords:** Adiponectin, Chinese, Indian, Malaysia, Diabetes Mellitus, Type 2, Motor Activity, Diet Records

## Abstract

**Background:**

The Malaysian Health and morbidity Survey (2006) reported the highest prevalence of type 2 diabetes mellitus (T2DM) among the Indian population compared to the Malay and Chinese populations. Many studies have supported the important role of adiponectin in insulin-sensitizing, which is associated with T2DM. These studies have raised a research question whether the variation in prevalence is related to the adiponectin concentrations or the lifestyle factors.

**Objectives:**

The purpose of this study is to determine whether the adiponectin concentrations differ between the Malay, Chinese and the Indian populations with T2DM. It is to investigate the association of adiponectin concentrations with ethnicity, dietary intake and physical activity too.

**Materials and Methods:**

In this cross-sectional study, a total of 210 T2DM patients with mean (SD) age of 56.73 (10.23) years were recruited from Penang, Malaysia. Data on demographic background, medical history, anthropometry (weight, height, visceral fat, percentage of body fat and waist circumference), dietary intake (3 days 24 hours diet recall) and physical activity (International Physical Activity Questionnaire) were obtained accordingly. Plasma adiponectin and routine laboratory tests (fasting blood sugar, HbA1c, total cholesterol, LDL, HDL and triglyceride) were performed according to standard procedure.

**Results:**

After adjustment for physical activity and dietary intakes, the Indian population had significantly lower adiponectin concentrations (P = 0.003) when compared with the Malay and the Chinese populations, The Indian population also had significantly higher value of HbA1c (P = 0.017) and significantly lower HDL (P = 0.013). Plasma adiponectin concentrations was significantly associated with ethnicity (P = 0.011), dietary carbohydrate (P = 0.003) and physical activity total MET score (P = 0.026), after medical history, age, sex, total cholesterol and visceral fat adjusted. However, dietary carbohydrate and physical activity did not show significantly difference among the various ethnic groups.

**Conclusions:**

In conclusion, lower concentration of adiponectin in the Indian population when compared with the Malay and the Chinese populations is not associated with lifestyle factors. The possibility of adiponectin gene polymorphism should be discussed further.

## 1. Background

Diabetes mellitus is a serious cause for premature illness and mortality throughout the world(1). It is estimated that 6.4% of the world’s adult population, equal to 285 million people has been diagnosed withdiabetes. By the year 2030, the number is expected to increase to 438 million, corresponding to 7.8% of the world’s adult population (2). In Malaysia, the Ministry of Health carried out the first National Health and Morbidity Survey (NHMS) in 1986. It was reported 6.3% of adults older than 35 years had been diagnosed as diabetic (3). Ten years later, the National Health and Morbidity Survey II revealed that the prevalence of diabetes mellitus among adults of age ≥ 30 years old had increased to 8.3% (4), and increased again to 11.6% after another ten years, in 2006. The highest prevalence was shown among the Indian population with 19.9%, which was almost double that of other major races, followed by the Malay with 11.9% and the Chinese with 11.4% of their populations (5).

High energy intake, a sedentary lifestyle and age are among the key factors in developing T2DM (6). Lifestyle intervention incorporating diet consultation and education, with emphasis on exercise, had been shown as the most effective method to deal with T2DM (7). It works well, with effective glucose control occurring within days (8), as well as lowering HbA1c significantly (9). Recently, the diabetes research has focused on studying the possible role of genetic mutation and related protein concentrations on development of diabetes. Adiponectin, an adipocyte-specific gene product consists of 244 amino acids and abundantly present in the bloodstream, has been reported to be efficient in lowering blood glucose among T2DM patients and improve insulin sensitivity (10). Numerous studies have shown that concentration of plasma adiponectinis increased, decreased or is unchanged by dietary management (11-13) and physical activity levels (14-16). These contradicting outcomes reflect the fact that previous studies consisted of respondents with diverse physiological and pathological circumstances. In addition, different ethnic groups with their unique lifestyle habits may lead to discordant findings, as concentrations of plasma adiponectin has been shown to be significantly different among ethnic groups, where the Indian population has a significantly lower concentrations of plasma adiponectin and higher insulin resistance, compared to the Chinese and the Malay populations (17). To our knowledge, the association between plasma adiponectin concentrations with dietary intake and physical activity in multiethnic T2DM patients from Malaysia, with different lifestyle habits and genetic background, has not been previously reported.

## 2. Objectives

To investigate the lifestyle and genetic components involved in T2DM, among the multiethnic population of Malaysia with T2DM.

## 3. Patients and Methods

This is a cross-sectional study with non-probability convenience sampling method. It was carried out at Sungai Bakap Hospital, Penang. The study group comprised of 210 Malaysian adult respondents. Respondents were T2DM adults without insulin treatment. Respondents on thiazolidinediones treatment, type 1 diabetes mellitus and pregnant woman were excluded. The study protocol was reviewed and approved by the ethics committee of Universiti Sains Malaysia. Formal permission to conduct the study in the chosen hospital was obtained from the Ministry of Health, Malaysia. A written informed consent was obtained from the respondents before participation. Respondents were also asked to complete a socio demographic questionnaire, included age, sex, educational level, household income per month and occupation. The medical history of patients includes family history, duration of diabetes and smoking habit.

### 3.1. Anthropometric Measurements

All of the anthropometric measurements were performed according to standard procedures, before breakfast, with the respondents barefooted and minimal clothing. Body weight and height were measured by the same investigator using Fat Analyzer Scale Model HBF-356 (Omron, Japan) and wall-mounted microtoise tape (Seca bodymeter 208, Hamburg, Germany) to the nearest 0.1 kg and 0.1 cm, respectively. Body Mass index classification was based on World Health Organization (WHO) 2004 (18). The waist was measured immediately above the iliac crest while the hip was recorded at the widest part of the hips (19). The cut-off point of the waist-hip ratio (WHR) for males and female were 0.9 and 0.8 respectively, to define as central adiposity (20). Body fat and visceral fat were measured using Body Fat Analyzer Scale Model HBF-356 (Omron, Japan). Subjects stood barefooted on the scale and the arm extended straight out 90° to the body. The Omron body fat analyzer scale estimates the body fat percentage by the bioelectrical impedance (BI) method. Whereas, levels of visceral fat were calculated using Omron’s analysis of CT scans.

### 3.2. Dietary Intake

Dietary intake was assessed by using three days 24 hours dietary recall, which appears optimal for estimating energy compared to 24 hours diet recall (18).All the respondents were asked to recall all of the food and drink consumed on three nonconsecutive days including 1 weekend and 2 weekdays. It was carried out with extensive probing, preferably with tableware items such as bowls, dishes, spoons and glasses in commonly-used sizes, food models and pictures of common foods were used to assess food intake to enhance the portion sizes and their respective weight estimations (21). Given information were carefully checked in order to avoid forgotten or misreported data. Energy and nutrient intakes for the 3 days were determined with computer software by using Nutritionist Pro software (Axxya Systems, United States). The means of these values were used for analyzing.

### 3.3. Physical Activity

The last-7-day, short form, self-administered version of the International Physical Activity Questionnaire (IPAQ), was used to collect self-reported physical activity data. The IPAQ used MET energy expenditure estimation from the compendium of physical activities to code physical activity by intensity. The total physical activity MET-minute/week was then computed by summing the walking, moderate and vigorous MET-minute/week scored. The scores were then categorized into low, moderate and vigorous physical activity level according to the IPAQ categorical score (22).

### 3.4.Blood Collection

Study respondents fasted for at least 8 hours prior to blood taking. 15 ml blood was collected into two EDTA (ethylene-diamine-tetra-acetate) tube and immediately inverted gently 5 times. About 5 ml of blood was collected for the analysis of plasma adiponectin. The fresh blood was spun in a centrifuge (Hettich, Universal 320, Germany), at 3500 rpm for 15 minutes. Plasma adiponectin was analyzed by using one-step human adiponectin immunoassay kit (Millipore Corporation, USA). The sensitivity of the kits was 1.5 ng/mL, whereas, the inter-assay and intra-assay coefficient were 2.4% - 8.4% and 1.0% - 7.4%, respectively. Analysis of the adiponectin was carried out at the hematology laboratory Universiti Sains Malaysia at room temperature. Whereas, another 10 ml of collected blood was used to measure fasting blood sugar (FBS), HbA1c and lipid profile such as total-cholesterol, triglyceride, low-density lipoprotein (LDL) and high density lipoprotein (HDL). Principle measurements for fasting blood sugar and HbA1c were UV hexokinase and high performance liquid chromatography (HPLC) respectively, while principle measurement for HDL and LDL was homogenous, in addition, principle measurement for total-cholesterol and triglyceride was enzymatic. Percentage of HbA1c analyzed by Bio-Rad D10 Hemoglobin A1c Analytical, whereas the rest of the tests were analyzed by Chemistry Analyzer Unicel DXC 600.

### 3.5. Statistical Analyses

All statistical analyses were performed by using Statistical Package for Social Sciences SPSS version 19 (SPSS Inc., IBM). A 2-tailed value of P < 0.05 was considered significant. Distribution of the data was assessed by descriptive analysis. Each variable was examined for normality of frequency distribution based on the histogram. Normally distributed data were expressed as mean (SD) and skewed data were expressed as medium (Interquartile Range or IqR). Pearson test was conducted to determine association between plasma adiponectin concentration with anthropometric measurements, biochemical results, dietary intake and physical activity MET score. Meanwhile, One-way Analysis of Variance (ANOVA) and bonferroni test were applied to compare mean of plasma adiponectin concentration in ethnic groups and levels of physical activity. The stepwise general linear regression analyses were conducted to study association of adiponectin concentration with ethnicity, dietary intake and physical activity of study subjects, with confounders adjusted. The variables with P < 0.25 in simple linear regression analyses were selected as confounders. Before performing general linear regression, dietary intake was energy adjusted (23). The Analysis of Covariance (ANCOVA) was applied to compare the plasma adiponectin concentration between ethnicity with relation to their dietary intake and physical activity. A 2-tailed value of p<0.05 was considered significant.

## 4. Results

Demographic characteristics of the study variables for the Malay, Chinese and the Indian populations are shown in Table 1. A total of 210 T2DM patients with the mean (SD) age of 56.73 (10.23) years, were involved in this study, ranging from 31 to 78 years old. The Indian respondents were significantly younger compared to the Chinese respondents. However, there was no significant difference among the sex ratios between the ethnic groups. Majority of the respondents were married women (56.2%) and housewives (50%) and had low educational levels (39.5%).

**Table 1.** Demographic Characteristics of the Study Variables (n = 210; 70 Malay, 70 Chinese and 70 Indians)

Characteristic	Malay	Chinese	Indian	Total	P value
**Age, y, Mean ± SD** ^a^	56.70 ± 10.21	59.77 ± 10.01	53.07 ± 8.28	56.37 ± 10.23	< 0.001 ^b^
**Sex** **, No. (%)**					0.873 ^c^
Male	29 (13.8)	31 (14.8)	32 (15.2)	92 (43.8)	
Female	41 (19.5)	39 (18.6)	38 (18.1)	118 (56.2)	
**Marital status** **, No. (%)**					0.543 ^c^
Single	3 (1.4)	1 (0.5)	2 (1.0)	6 (2.9)	
Married	66 (31.3)	69 (32.9)	66 (31.4)	201 (95.6)	
Widow/ widower	1 (0.5)	0 (0.0)	2 (1.0)	3 (1.5)	
**Job** **, No. (%)**					0.021 ^c^
Housewife	33 (15.7)	38 (18.1)	34 (16.2)	105 (50.0)	
Retired	15 (7.1)	17 (8.1)	8 (3.8)	40 (19.0)	
Self-employed	2 (1.0)	3 (1.4)	4 (1.9)	9 (4.3)	
Civil servant	11 (5.2)	2 (1.0)	4 (1.9)	17 (8.1)	
**Education Level** **, No. (%)**					0.048 ^c^
Non-educated	11 (5.2)	24 (11.4)	13 (6.2)	48 (22.8)	
Primary	29 (13.8)	17 (8.1)	27 (12.9)	73 (34.8)	
Secondary	26 (12.4)	28 (13.3)	29 (13.8)	83 (39.5)	
Tertiary	4 (1.9)	1 (0.5)	1 (0.5)	6 (2.9)	

^a^SD: Standard deviation

^b^One way Analysis of Variance

^c^Bonferroni test; 2 Chi-square test

Descriptive statistics of the study variables is shown in Table 2. A majority of the respondents had overall (63.4%) and central adiposity (83.8%), as well as hyperlipidaemia (66.2%). The Indian respondents showed significantly higher value of HbA1c compared to the Chinese (P = 0.017) respondents. The Indian respondents also showed significantly lower plasma adiponectin concentration (P = 0.010) and HDL (P = 0.013) compared to the Malay respondents. By distributing into percentage, dietary carbohydrate, protein and fat intake of respondents were 60%, 16% and 24% respectively. The Chinese respondents showed significantly higher dietary protein (P = 0.002) and fiber intake (P = 0.001). However, only 3% of total respondents achieved the target of dietary fiber intake recommended by Medical Nutrition Therapy Diabetes Division (2005) ( 24 ). No significant difference in physical activity was observed between the three ethnicities.

**Table 2.** Descriptive Statistics of the Study Variables (n = 210; 70 Malay, 70 Chinese and 70 Indians)

Variables	Total, Mean ± SD ^a^	Malay, Mean ± SD	Chinese, Mean ± SD	Indian, Mean ± SD	F ratio	P value ^b^
**Anthropometry**						
BMI **^a^**, kg/m ^2^	27.50 ± 5.03	28.17 ± 5.51	26.54 ± 4.03	27.78 ± 5.34	2.02	0.135
Body fat status, %	32.23 ± 7.10	32.30 ± 7.24	31.17 ± 6.31	33.21 ± 7.64	1.46	0.234
WC **^a^**, cm	93.56 ± 11.49	94.17 ± 12.69	91.90 ± 9.01	94.61 ± 12.38	1.13	0.326
Waist hip ratio	0.91 ± 0.07	0.91 ± 0.08	0.90 ± 0.06	0.92 ± 0.06	0.58	0.561
Visceral fat level	13.29 ± 5.92	14.10 ± 6.42	12.06 ± 4.81	13.70 ± 6.27	2.37	0.096
**Blood ** **Result**						
Adiponectin, μg/ml	6.01 ± 3.71	6.85 ± 4.66 a	6.21 ± 3.62	4.98 ± 2.22 b	3.54	0.010
FBS **^a^**, mmol/L	10.29 ± 5.81	10.48 ± 3.97	9.25 ± 3.48	11.15 ± 8.51	2.13	0.122
HbA1c, %	8.52 ± 1.73	8.53 ± 1.69	8.10 ± 1.65 c	8.93 ± 1.78 d	4.15	0.017
TC **^a^**, mmol/L	5.06 ± 1.15	5.20 ± 1.13	5.06 ± 1.11	4.94 ± 1.20	0.91	0.403
LDL **^a^**, mmol/L	3.29 ± 0.90	3.33 ± 0.96	3.25 ± 0.90	3.28 ±0.84	0.14	0.868
HDL **^a^**, mmol/L	1.00 ± 0.35	1.05 ± 0.50 e	1.03 ± 0.29	0.91 ± 0.16 f	4.43	0.013
TG **^a^**, mmol/L	1.89 ± 1.20	2.06 ± 1.30	1.84 ± 1.09	1.78 ± 1.20	1.10	0.334
**Dietary Intake**						
Calorie, kcal	1647 ± 564.44	1583 ± 477.40	1714 ± 582.30	1643 ± 623.93	0.95	0.389
Protein, g	67.40 ± 22.93	63.24 ± 18.70 g	75.37 ± 26.37 h	63.58 ± 21.22 j	6.69	0.002
CHO **^a^**, g	240.73 ± 92.63	236.97 ± 83.59	238.36 ± 87.21	246.87 ± 106.49	0.43	0.654
Fat, g	46.92 ± 23.41	44.83 ± 20.60	50.56 ± 26.83	45.38 ± 22.28	1.28	0.281
Fiber, g	7.51 ± 0.35	6.40 ± 0.51 k	9.33 ± 0.71 m	6.78 ± 0.51 n	7.46	0.001
**Physical Activity**						
MET score	3352.53 ± 4251.35	3609.78 ± 4354.71	2595.67 ± 2801.41	3789.90 ± 5200.00	1.57	0.201

^a^Abbreviations: SD, Standard deviation; BMI, Body mass index; WC, Waist circumference; FBS, Fasting blood sugar; TC, Total cholesterol; LDL, Low density lipoprotein; HDL, High density lipoprotein; TG, triglyceride; CHO, Carbohydrate

^b^Analysis of Variance, Bonferroni test was applied. Test shows: a vs b, P < 0.05; c vs d, P < 0.05; e vs f, P < 0.05. g vs h, P < 0.01; h vs j, P < 0.01; k vs m, P = 0.001; m vs n, P < 0.01

Pearson’s correlation coefficient was applied to determine the relationship between plasma adiponectin concentrations with the anthropometric measurements, biochemical results, dietary intake and the physical activity and it is presented in Table 3. Plasma adiponectin concentrations had a significant inverse relationship with physical activity MET score, whereas it had significant positive correlation with HDL. No statistically significant correlation was observed between plasma adiponectin with anthropometric measurements and dietary intake. The findings from the simple linear regression analyses were further explored using general linear regressions. The outcome is presented in Table 4. An inverse relationship was shown between plasma adiponectin concentrations with physical activity MET score (P = 0.003) and dietary carbohydrate intake (P = 0.026), after medical history, age, sex, total cholesterol and visceral fat adjusted. A One standard deviation increase in total MET score, lead to 0.203 standard deviation decreased in plasma adiponectin concentrations. Whereas, a one standard deviation increase in carbohydrate intake will lead to 0.150 standard deviation decrease in plasma adiponectin concentrations. A higher intake of carbohydrates and higher physical activity levels will lead to a lower concentration of plasma adiponectin. Results from general linear analyses also revealed a significant difference in plasma adiponectin concentrations within the ethnicity (P = 0.011). The Indian respondents had 0.171 µg/mL lower plasma adiponectin concentrations as compared to the Malay respondents.No significant association was shown in plasma adiponectin concentrations between the Chinese and Indian respondents, or the Chinese and Malay respondents. Dietary fat, protein and fiber intake were shown to have no significant association with plasma adiponectin concentrations.

**Table 3.** Pearson’s Correlation Coefficients Between Plasma Adiponectin Concentrationwith Anthropometric Measurements, Biochemical Results, Dietary Intake and Physical Activity (n = 210)

Variables	Adiponectin Concentration	
	R	P value
**Anthropometric Measurements**		
Body mass index, kg/m ^2^	-0.02	0.810
Percentage of fat, %	0.07	0.349
Waist circumference, cm	-0.06	0.367
Waist hip ratio	-0.08	0.267
Visceral fat, level	0.09	0.217
**Biochemical Results**		
Fasting blood sugar, mmol/L	0.04	0.569
HbA1c, %	-0.06	0.427
Total cholesterol, mmol/L	0.08	0.229
Low density lipoprotein, mmol/L	-0.003	0.961
High density lipoprotein, mmol/L	0.21	0.002 ^a^
Triglyceride, mmol/L	-0.03	0.703
**Dietary Intake**		
Calorie, mjoule	-0.14	0.054
Adjusted dietary carbohydrate, g	-0.12	0.056
Adjusted dietary protein, g	-0.05	0.458
Adjusted dietary fat, g	-0.11	0.102
Adjusted dietary fiber, g	-0.03	0.671
**Physical Activity **		
MET score	-0.18	0.007 ^b^

^a^is significant at the 0.01 level

^b^is significant at the 0.05 level

**Table 4.** Relationship Between Plasma Adiponectin Concentration with Ethnicity, Dietary Intake and Physical Activity for Type 2 Diabetes Patients Among Different Ethnic Groups (n = 210)

Variables	Simple Linear Regression		General Linear Regression	
	b ^a^(95%, CI)	P value	b ^b^(95%, CI)	P value
**Ethnic, Malay and Indian**	0.238 (0.649, 3.077)	0.003	0.171 (0.311, 2.375)	0.011
**Total MET Score**	-0.184 (0.000, 0.000)	0.007	-0.203 (0.000, 0.000)	0.003
**Adjusted Dietary Carbohydrate**	-0.132 (-0.012, 0.000)	0.056	-0.150 (-0.013, -0.001)	0.026

^a^Crude regression coefficient

^b^Adjusted regression coefficient for medical history, age, sex, total cholesterol and visceral fat Stepwise multiple linear regression method applied. Model assumptions are fulfilled. There were no interactions among independent variables. No multicollinearity detected. Coefficient of determination (R²) = 0.153

A comparison of adiponectin concentrations between the three ethnicities in relation to theirdietary intake and physical activity by using ANCOVA is shown in Table 5. Plasma adiponectin concentrations showed significant differences when compared between ethnic groups (P = 0.010). Moreover, after adjustment of lifestyle factors such as dietary fat, protein, carbohydrate, fiber intake and physical activity MET score, the mean of plasma adiponectin concentrations was still significantly different among ethnic groups (P = 0.003).

**Table 5.** Comparison of Adiponectin Concentration between Ethnicity with Relation to Their Dietary Intake and Physical Activity for Type 2 Diabetes Patients (n = 210)

	Adiponectin [Table-fn fn3043] Concentration, μg/mL		
Groups	Adj. Mean ^a^ (95% CI)	F-ratio ^a^	P value ^a^
**Ethnicity**		9.32	0.003
Malay ^b^	7.202 (6.298, 8.106)		
Chinese	6.687 (5.799, 7.575)		
Indian ^b^	5.597 (4.651, 6.544)		

^a^Total MET score, fat, protein, carbohydrate and fiber adjusted;

^b^Test shows: Malay vs. Indian P < 0.05;

## 5. Discussion

In this present study, the Indian respondents showed significantly lower plasma adiponectin concentrations compared to the other ethnic groups. This is consistent with a study done in Singapore (17). The Indian respondents also showed the highest prevalence of central adiposity.This could be one of the reasons the Indian respondents had the lowest plasma adiponectin concentrations as central adiposity reported to have a strong relationship with the plasma adiponectin concentrations (25). Furthermore, the Chinese respondents showed a statistically significant higher intake of dietary protein and fiber. Increasing dietary protein from 15% to 30% of total calorie intake at the expense of carbohydrate increasing the integration of insulin concentration and reducing the 24 hours integration of glucose concentrations (26). This might be one of the reasons the Chinese respondents showed a better control in T2DM with the lowest fasting blood sugar and HbA1c, compared to the Indian and the Malay respondents. A significant inverse correlation between plasma adiponectin concentrations with dietary carbohydrate intake was shown. It is consistent with the studies from Germany (23), California (24) and Boston in T2DM patients (27). A previous study conducted among T2DM patients and non-T2DM patients demonstrated that Interleukin concentrations decreased and plasma adiponectin concentrations increased after consumption of high carbohydrate and high fiber meals, however, contrasting result was shown after consumption of high carbohydrate and low fiber meals (16). In the present study, percentage of total carbohydrate intake was 60%. It is consistent with the guideline from the Ministry of Health Malaysia. However, only 3% of respondents achieved the target of dietary fiber intake recommended by Ministry of Health Malaysia. This may explain the cause of low plasma adiponectin concentrations despite respondent being on target with the guideline recommendation of total carbohydrate intake. The outcome of the study revealed the importance of carbohydrate’s quality, as compared to the overall carbohydrate intake. Carbohydrate with low glycaemic index should be studied further.

Plasma adiponectin concentrations also demonstrated a significant inverse relationship with levels of physical activity, with confounder adjusted. It is in accordance with the studies from United Kingdom (15), Japan (28) and United States (29). Previous studies showed that plasma adiponectin concentrations will be down regulated (15) when physical activity induces the sensitivity of insulin (28). Long-term physical activity intervention shown to increase the receptors of adiponectin and reduce the levels of adiponectin concentrations (30). In addition, physical activity might change the level of adiponectin in isoform, especially the high molecular weight (HMW) isoform, which was not studied in this paper(31). The ratio of adiponectin multimer isoforms may be changed by physical activity, thereby the biological active form of plasma adiponectin may be increased (30). This outcome showed that studies on isoforms of plasma adiponectin would be more accurate as compared to total adiponectin concentrations only. This paper showed that hypoadiponectinemia seen in the Indian respondents was independent of lifestyle factors, as dietary carbohydrates and physical activity did not show any significant differences among the various ethnic groups. The Indian respondents also had a significantly higher percentage of HbA1c and significantly lower HDL concentrations. In conclusion, lower concentration of plasma adiponectin in the Indian respondents compared to the Malay and the Chinese respondents is not associated with lifestyle factors. The possibility of adiponectin gene polymorphism should be studied further.

For this present study, convenience sampling method might introduce selection bias (32), and it is unable be used to ensure approximately unbiased estimators of population quantities or provide associated measures of precision (16). Another limitation is the possibility of misreporting of physical activity data. Study respondents may have over reported their physical activity levels in order not to embarrass themselves in front of a healthcare professional. It is suggested that further studies use an accelerometer to measure physical activity level in order to directly detect frequency of human motion (33). Besides this, a study on HMW and other isoforms of adiponectin associated with T2DM needs to be done, as HMW has potential roles in directly sensitizes the body to insulin (25), glucose (34) and lipid homeostasis secretion from adipose tissue. Studying HMW would be more accurate as compared to using total adiponectin levels.

## References

[1] Sayyad Mehmood G, Shahani Arjan K, Pal Sanju, Shirodkar Jyoti A, Gopal G (2011). Computer Simulation Modelling for the Prevention of Diabetes Mellitus (Prameha).. J Appl Sci..

[2] Wild S, Roglic G, Green A, Sicree R, King H (2004). Global prevalence of diabetes: estimates for the year 2000 and projections for 2030.. Diabetes Care..

[3] (1986-1987). Diabetes Mellitus. National Health Morbidity Survey (NHMS I)..

[4] (1996). National Health Morbidity Survey (NHMS II)..

[5] (2006). National Health Morbidity Survey (NHMS III)..

[6] Bantle John P, Wylie-Rosett Judith, Albright Ann L, Apovian Caroline M, Clark Nathaniel G, Franz Marion J (2008). Nutrition recommendations and interventions for diabetes: a position statement of the American Diabetes Association.. Diabetes Care..

[7] Boule NG, Haddad E, Kenny GP, Wells GA, Sigal RJ (2001). Effects of exercise on glycemic control and body mass in type 2 diabetes mellitus: a meta-analysis of controlled clinical trials.. JAMA..

[8] Nakano Y, Tobe T, Choi-Miura NH, Mazda T, Tomita M (1996). Isolation and characterization of GBP28, a novel gelatin-binding protein purified from human plasma.. J Biochem..

[9] Berg AH, Combs TP, Du X, Brownlee M, Scherer PE (2001). The adipocyte-secreted protein Acrp30 enhances hepatic insulin action.. Nat Med..

[10] Stirban A, Negrean M, Stratmann B, Gotting C, Salomon J, Kleesiek K (2007). Adiponectin decreases postprandially following a heat-processed meal in individuals with type 2 diabetes: an effect prevented by benfotiamine and cooking method.. Diabetes Care..

[11] Yang WS, Lee WJ, Funahashi T, Tanaka S, Matsuzawa Y, Chao CL (2002). Plasma adiponectin levels in overweight and obese Asians.. Obes Res..

[12] Khoo CM, Sairazi S, Taslim S, Gardner D, Wu Y, Lee J (2011). Ethnicity modifies the relationships of insulin resistance, inflammation, and adiponectin with obesity in a multiethnic Asian population.. Diabetes Care..

[13] Riera-Guardia N, Rothenbacher D (2008). The effect of thiazolidinediones on adiponectin serum level: a meta-analysis.. Diabetes Obes Metab..

[14] (2004). Appropriate body-mass index for Asian populations and its implications for policy and intervention strategies.. Lancet..

[15] (1988-1994). The Third National Health and Nutrition Examination Survey (NHANES III)..

[16] Forster JJ (2001). Sample surveys: nonprobability sampling..

[17] Hsieh CJ, Wang PW, Liu RT, Tung SC, Chien WY, Chen JF (2005). Orlistat for obesity: benefits beyond weight loss.. Diabetes Res Clin Pract..

[18] Ma Y, Olendzki BC, Pagoto SL, Hurley TG, Magner RP, Ockene IS (2009). Number of 24-hour diet recalls needed to estimate energy intake.. Ann Epidemiol..

[19] Suzana S, Noor Aini MY, Shanita SN, Rafidah G, Roslina A (2009). Atlas of food exchanges and portion sizes..

[20] IPAQ Research Committee (2005). Guidelines for the data processing and analysis of the International Physical Activity Questionnaire..

[21] Malaysia Dietitians’ Association (2005). Medical nutrition therapy guidelines for type 2 diabetes.. Ministry of Health, Kuala Lumpur, Malaysia..

[22] Margetic S, Gazzola C, Pegg GG, Hill RA (2002). Leptin: a review of its peripheral actions and interactions.. Int J Obes Relat Metab Disord..

[23] Pischon T, Girman CJ, Rifai N, Hotamisligil GS, Rimm EB (2005). Association between dietary factors and plasma adiponectin concentrations in men.. Am J Clin Nutr..

[24] Qi L, Rimm E, Liu S, Rifai N, Hu FB (2005). Dietary glycemic index, glycemic load, cereal fiber, and plasma adiponectin concentration in diabetic men.. Diabetes Care..

[25] Kadowaki T, Yamauchi T (2005). Adiponectin and adiponectin receptors.. Endocr Rev..

[26] Gannon MC, Nuttall FQ (2006). Control of blood glucose in type 2 diabetes without weight loss by modification of diet composition.. Nutr Metab (Lond)..

[27] Schulze MB, Rimm EB, Shai I, Rifai N, Hu FB (2004). Relationship between adiponectin and glycemic control, blood lipids, and inflammatory markers in men with type 2 diabetes.. Diabetes Care..

[28] Yatagai T, Nishida Y, Nagasaka S, Nakamura T, Tokuyama K, Shindo M (2003). Relationship between exercise training-induced increase in insulin sensitivity and adiponectinemia in healthy men.. Endocr J..

[29] Nemet D, Wang P, Funahashi T, Matsuzawa Y, Tanaka S, Engelman L (2003). Adipocytokines, body composition, and fitness in children.. Pediatr Res..

[30] O'Leary VB, Jorett AE, Marchetti CM, Gonzalez F, Phillips SA, Ciaraldi TP (2007). Enhanced adiponectin multimer ratio and skeletal muscle adiponectin receptor expression following exercise training and diet in older insulin-resistant adults.. Am J Physiol Endocrinol Metab..

[31] Tsao TS, Tomas E, Murrey HE, Hug C, Lee DH, Ruderman NB (2003). Role of disulfide bonds in Acrp30/adiponectin structure and signaling specificity. Different oligomers activate different signal transduction pathways.. J Biol Chem..

[32] Jafary FH, Aslam F, Mahmud H, Waheed A, Shakir M, Afzal A (2005). Cardiovascular health knowledge and behavior in patient attendants at four tertiary care hospitals in Pakistan--a cause for concern.. BMC Public Health..

[33] Bravata DM, Smith-Spangler C, Sundaram V, Gienger AL, Lin N, Lewis R (2007). Using pedometers to increase physical activity and improve health: a systematic review.. JAMA..

[34] Yamauchi T, Kamon J, Minokoshi Y, Ito Y, Waki H, Uchida S (2002). Adiponectin stimulates glucose utilization and fatty-acid oxidation by activating AMP-activated protein kinase.. Nat Med..

